# Teaching and Embracing Uncertainty in Medicine and Psychiatry: A Paradigm Shift

**DOI:** 10.1192/j.eurpsy.2026.10955

**Published:** 2026-07-31

**Authors:** T. Lorenzetto, A. Platini, A. E. M. Bobbio, N. F. De Luca, G. Volta, F. Gavelli, M. Bellan, P. Zeppegno, E. Gambaro, R. Calati, M. Pirisi, F. Turolla, C. Gramaglia

**Affiliations:** 1 Università del Piemonte Orientale, Novara, Italy; 2 Translational Medicine, Università del Piemonte Orientale, Novara; 3 Università Milano Bicocca, Milano, Italy

## Abstract

**Introduction:**

Uncertainty is an inevitable dimension of both medical and psychiatric practice and before these, of human nature. While early research showed that tolerance for uncertainty (UT) develops gradually with experience, traditional medical education has often reinforced an illusion of certainty. Recent studies have positioned UT as a critical competency for medical graduates and in psychiatry it is especially relevant given the complexity of diagnosis, prognosis and treatment. Validated measurement tools and educational interventions have been developed to strengthen UT. Importantly, the New England Journal of Medicine described the acceptance of uncertainty as “the next medical revolution,” underlining the cultural significance of this paradigm shift.

**Objectives:**

To explore medical students’, residents’ and clinicians’ perceptions of uncertainty, identify factors influencing UT and evaluate implications for medical and psychiatric education.

**Methods:**

A preliminary survey study is being conducted among medical students and residents. The questionnaire was developed specificallyfor this study, based on constructs identified in the literature on uncertainty in medicine. It collects sociodemographic, educational and experiential data, together with self-reported attitudes toward uncertainty. Data collection is ongoing. In parallel, a narrative review of recent literature (2018–2025) was performed, focusing on theoretical frameworks, survey tools and educational interventions in medicine and psychiatry.

**Results:**

Results are pending. Current evidence from the literature indicates that while several uncertainty tolerance scales exist, none fully cover all domains of validity and their reliability is weaker in students than in physicians. Educational interventions (including problem-based learning, simulation, reflective practice and medical humanities) generally improve UT in cognitive and behavioral domains, although negative emotional reactions may persist.

**Conclusions:**

Medical and psychiatric education are undergoing a cultural shift: from concealing uncertainty to openly addressing and teaching it. Tolerating uncertainty should be reframed not merely as an individual skill but as a systemic educational objective. As emphasized in the NEJM perspective, embracing uncertainty is not only necessary but represents an authentic “revolution” in preparing future physicians and psychiatrists for the complexity of contemporary healthcare.

**Disclosure of Interest:**

None Declared

